# PRESIDENTIAL SYMPOSIUM: THE TIES THAT BIND: THE INFLUENCE OF SOCIAL MEDIA AND TECHNOLOGY IN THE LIVES OF OLDER ADULTS

**DOI:** 10.1093/geroni/igz038.766

**Published:** 2019-11-08

**Authors:** Tamara A Baker, Lewina O Lee

**Affiliations:** 1 University of Kansas, Lawrence, Kansas, United States; 2 Boston University School of Medicine, Boston, Massachusetts, United States

## Abstract

Data show that seven out of every ten adults, over the age of 50, own a smartphone, with one out of ten owning a tablet. While traditional activities dictate the use of technology among this cohort, there is growing evidence that adults similarly use devices to also manage their medical care and to learn online. This increase has guided scholars in recognizing the utility of technology from designing interventions to understanding how technology may serve as a barrier and/or facilitator to one’s general well-being. This symposium features four presentations from nationally recognized scholars that will expand traditional perspectives on technology use, and how it influences social ties among older adults. Dr. Charness will examine the population-level trends in social network use by aging adults and discuss a recent CREATE intervention study (PRISM), that used a computer-based platform to reduce social isolation and loneliness among older adults. Dr. Czaja will similarly present findings from CREATE, and other trials, on the access to and use of email, social media sites, and online support groups among older adults, and the resultant impact on social connectivity, loneliness and social support. Dr. Rogers will discuss technologies that currently exist (e.g., apps, mobile devices, social networking) or are being developed (e.g., robotics, telepresence, virtual reality) to support social engagement. Dr. Antonucci will examine aspects of new technologies and their influence on health and well-being, while underscoring the perspective that new and emerging technologies hold great promise in overcoming traditional barriers to maintaining social contact and exchange.

